# Subcision: A Further Modification, an Ever Continuing Process

**DOI:** 10.1155/2012/685347

**Published:** 2012-12-12

**Authors:** Mohanned A. Alsufyani, Mohammed A. Alsufyani

**Affiliations:** ^1^Collage of Medicine, King Saud University for Health Sciences, Riyadh 103053, Saudi Arabia; ^2^Department of Dermatology, Prince Sultan Military Medical City, Riyadh 11159, Saudi Arabia

## Abstract

Subcision is a surgical technique used mostly to manage depressed scars. Over time, many modifications to this surgical technique have been made by various surgeons in order to make it simpler and more effective. We report here a new technique that aims to combine the privilege of the prevention of penetrating the skin beyond the scar and maintaining a horizontal orientation, while taking the advantage of the ergonomics of having the dominant hand parallel to the skin surface and the cylindrical grip of a 3 cc syringe. The purpose of our technique is to make subcision more practical and easier for the surgeon.

## 1. Introduction 

Subcision is a surgical technique used mostly to manage depressed scars [1]. As described by S. Orentreich and N. Orentreich in 1995 [2], subcision aims to sever the fibrous attachments beneath the scar at the subdermal level to lift up the scar and induce the formation of connective tissues through normal physiological healing [1, 3]. This is achieved using a Nokor or hypodermic needle (needle gauge depends on the size of the scar). 

Over time, many modifications to this surgical technique have been made by various surgeons [1, 4] in order to make it more simpler and more effective. One modification made by N. Khunger and M. Khunger [1] was bending the needle at a 90-degree angle using artery forceps, the purpose for the angle created was to prevent the needle from penetrating the skin or causing damage to the dermis. Another modification made by Al Ghamdi was using a needle holder for the Nokor needle to maintain its horizontal orientation without the need for withdrawal from the entry point to visualize the orientation and redirect the needle [4].

We report here a new technique that further modifies Khunger's technique and aims to combine the privilege of both of the previously mentioned techniques, the prevention of penetrating the skin beyond the scar and maintaining a horizontal orientation, while taking the advantage of the ergonomics of having the dominant hand parallel to the skin surface and the cylindrical grip of a 3 cc syringe. The purpose of our technique is to make subcision more practical and easier for the surgeon.

## 2. Description of the Technique

**Figure 1:**
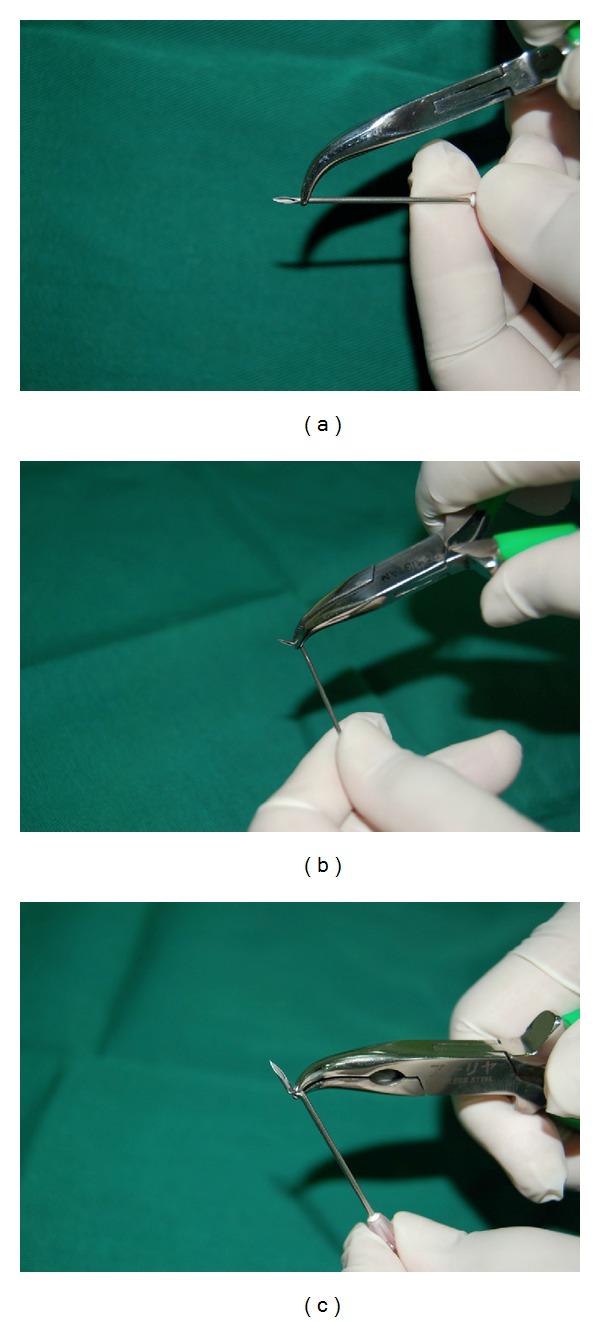
(a) The tip of the plier is positioned just proximal to the bevel of the needle. (b) The needle is bent with an upward motion till it reaches 90-degrees, creating the first angle. (c) The tip of the plier is moved 1-2 mm proximal to the first angle and a second 90-degree angle is created opposite to the direction of the first angle.

**Figure 2:**
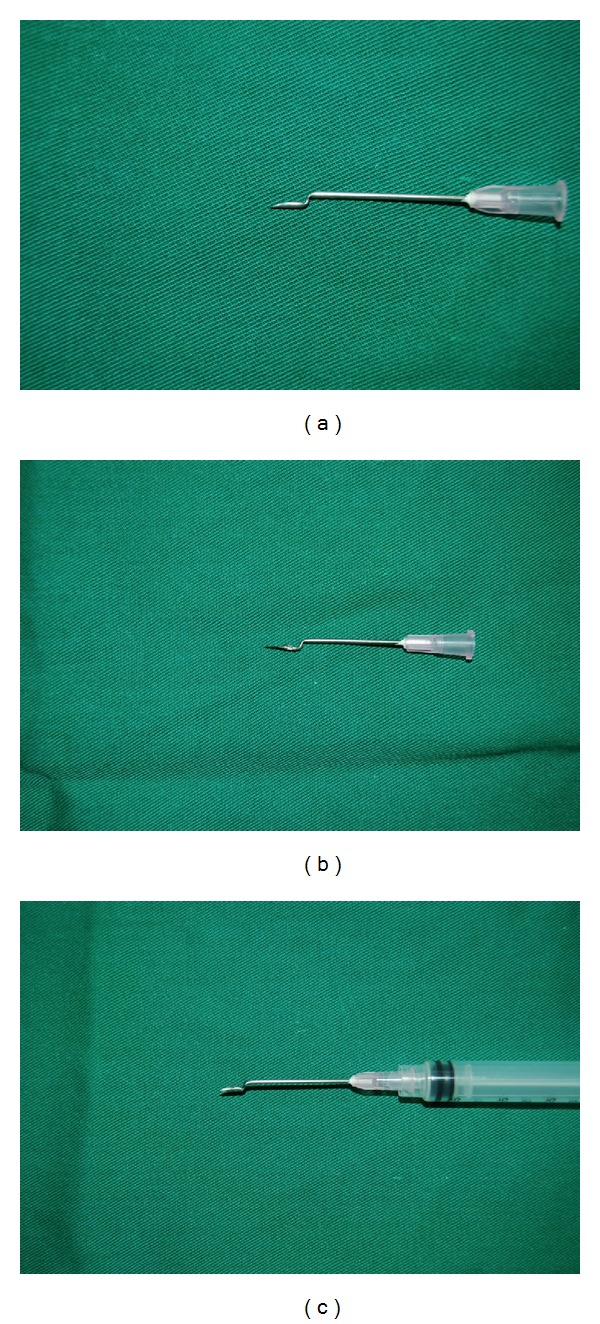
(a) The final shape of the hypodermic needle. (b) The same technique may be applied to a Nokor needle. (c) The modified hypodermic needle attached to a 3 cc luer lock syringe.

**Figure 3:**
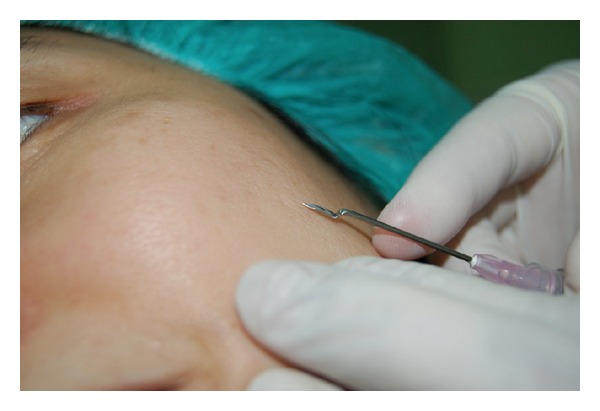
The parallel alignment of the needle to the skin surface conforms an ergonomic motion while serving the purpose of the final configuration of the needle, that is, preventing penetration of the skin beyond the scar and maintain a horizontal orientation of the needle tip.

The needle, 18G × 1.5 in. BD Nokor needle (Becton, Dickinson & Co.), USA or 18G × 1 in. KBM hypodermic needle, Japan, is held with the bevel facing up just proximal to it; using curved pliers or artery forceps, it is bended in an upright orientation, until it reaches a 90-degree angle. After that the forceps (or plier) is advanced 1-2 mm proximal to the first angle and a second 90-degree angle is created by bending the needle in a downward orientation, opposite to the direction of the first angle. The final shape of the hypodermic or Nokor needle resembles a two-step shape, with the first step having the bevel of the needle directed superiorly and has a parallel orientation to the skin surface, as shown in Figures 1(a)–1(c) and 2(a)–2(c). This final configuration of the needle allows the surgeon to take advantage of the first angle to serve its purpose for preventing the penetration of the skin beyond the scar. The shape of the needle also helps maintaining a horizontal orientation of the bevel of the needle (Nokor or hypodermic) as the practitioner can use the step between the two angles to guide him/her about the direction and orientation. The surgeon can benefit, as well, from the more ergonomic motion for subcising scars with the dominant hand maintained parallel to the skin surface as opposed to moving perpendicular to it, which exerts some stress on the wrist joint (Figure 3). Furthermore, the more ergonomic grip of the cylindrical body of a 3 cc syringe used to hold the bended needle offers an additional advantage to the subcision technique, compared to other awkward physical shapes, an artery forceps, for instance.

## 3. Discussion

Subcision is a helpful surgical technique that can be used to treat acne scars, depressed scars, wrinkles, striae, and cellulite, but not other types of scars such as boxcar or deep pitted scars [1, 3, 5].

The main principle of subcision is detachment of the epidermal layer from the fibrotic strands found in scars, which anchor the scar to the subcutaneous tissue [3]. A Nokor or hypodermic needle is introduced into the subdermal space parallel to the skin and moved back and forth and in a fanning motion to release the skin. The depression is lifted by this motion as well as by normal wound healing, through connective tissue formation and collagenization. Common complications of this technique include postoperative hematoma, pain, bruising, swelling, induration, and hyperpigmentation [1, 3, 6, 7].

Precautions should be taken to make sure sufficient anaesthesia is used for patient comfort and that the procedure is done with caution when performed near superficial nerves (e.g., in the preauricular region) [3, 6].

One obstacle in using subcision, especially with the Nokor needle, was rectified by Al Ghamdi. With the goal of maintaining a horizontal orientation, Al Ghamdi proposed that holding the Nokor needle with a needle holder gives control of the orientation without the need to withdraw from the point of entry and visualize the orientation [4]. Another issue with subcision is maintaining needle depth. This was addressed by N. Khunger and M. Khunger, who stated that using an artery forceps to bend the needle at a 90-degree angle, gives the surgeon depth control and thus prevents the needle from penetrating the skin and causing dermal damage [1]. After reading, with interest, N. Khunger and M. Khunger [1] technique, we experimented it on a few patients and found it to be very helpful in terms of preventing the needle from penetrating the skin, but we also felt that holding the needle perpendicular to the skin surface to keep the tip of the needle parallel to the skin surface, after having bent the needle at a 90-degree angle with the bevel of the needle directed superiorly, was rather difficult to manoeuvre and exerted some level of stress on the surgeon's wrist joint. With that kept in mind, we modified the technique by adding another 90-degree angle.

With our technique, we found that the first 90-degree angle has the advantage of preventing the needle from penetrating the skin and controlling the depth, as proposed by N. Khunger and M. Khunger [1]. But we also found that holding the needle perpendicular to the skin surface exerted some stress on the wrist joint and was somewhat physically awkward. So we added a second 90-degree angle, just proximal to the first one for two purposes. The first was to redirect the needle to be parallel to the skin surface, alleviating the physical awkwardness of the position when maintaining it perpendicular. The second reason is that the step existing between the two angles acts as a guide for maintaining a horizontal orientation. All of this while serving the purpose of the first 90-degree angle for preventing the needle from penetrating the skin beyond the scar and maintaining depth, as described by N. Khunger and M. Khunger Furthermore, we believe that the cylindrical shape and size of the 3 cc syringe has the advantage of a better ergonomic grip compared to that afforded by artery forceps. The needle, as with the original method of subcision for acne scars, is reused for treating the remainder of acne scars existing on the patient's face.

Few limitations do exist for this particular technique that is worthwhile mentioning. First, the technique uses large bore needles, that is, 18 gauge needles, so the issue of stability of a needle when using this particular technique with a smaller gauge needle, for instance, a 23 gauge, cannot be assured. Second, this technique was specifically developed for subcising acne scars. Using it for longer scars would be rather difficult, as the first created angle acts as a guard for the needle to limit its insertion beyond the bevel's length. Finally, since the technique involves twisting and turning of a needle that is not meant for this purpose, concern might rise regarding the strength of the needle at the created angles and whether or not there is risk of the tip breaking inside the skin. However, it is also worthwhile stating that the same twisting and turning of such needles with different gauges, for example, 20, 19, and 18, but with the angles created to the opposite direction of the technique we are reporting, was used in the creation of recipient sites for follicular unit transplantation in procedures of hair restoration surgeries that have been used by surgeons (including the second author, M. Alsufyani) performing such procedures for many years, and we have not found a report on the needle tip breaking inside the skin, despite of using the same needle for creating an average of 50–100 recipient sites. 

To conclude, all surgical techniques are developed and modified continuously over time with the sole purpose of achieving the best outcomes through easier, comfortable, and practical ways to allow the best final results with the least of complications and fastest recovery for the patient. In this paper, we exemplify that by taking a modified innovative surgical technique and modify it even further to make subcision a more simpler, effective, and physically ergonomic procedure. As time passes on, we are certain that more innovations on subcision will emerge to provide the practicing dermatologic surgeon with vast options of techniques to choose from each with what best fits his/her skills.
